# Nano-ZnO Catalyzed Multicomponent One-Pot Synthesis of Novel Spiro(indoline-pyranodioxine) Derivatives

**DOI:** 10.1155/2014/427195

**Published:** 2014-02-05

**Authors:** Harshita Sachdeva, Rekha Saroj, Diksha Dwivedi

**Affiliations:** Department of Chemistry, Faculty of Engineering and Technology, Mody Institute of Technology and Science, Lakshmangarh, Sikar, Rajasthan 332311, India

## Abstract

A simple catalytic protocol for the synthesis of novel spiro[indoline-pyranodioxine] derivatives has been developed using ZnO nanoparticle as an efficient, green, and reusable catalyst. The derivatives are obtained in moderate to excellent yield by one-pot three-component reaction of an isatin, malononitrile/ethylcyanoacetate, and 2,2-dimethyl-1,3-dioxane-4,6-dione in absolute ethanol under conventional heating and microwave irradiation. The catalyst was recovered by filtration from the reaction mixture and reused during five consecutive runs without any apparent loss of activity for the same reaction. The mild reaction conditions and recyclability of the catalyst make it environmentally benign synthetic procedure.

## 1. Introduction

Heterocyclic chemistry is one of the most complex and intriguing branches of organic chemistry and heterocyclic compounds constitute the largest and most varied family of organic compounds. Among heterocyclic compounds, indole derivatives exhibit a number of biological activities [1–8], for example, antimicrobial, anticonvulsant, antineoplastic, antiviral, antihypertensive, anti-inflammatory, and enzymatic inhibition activities dopaminergic agonist and so forth. In addition to substituted and condensed heterocycles, spiroindoles, with C-3 as spiro atom, have received considerable interest due to their strong biological activities [9–11].

Further, Meldrum's acid (2,2-dimethyl-1,3-dioxane-4,6-diones) is useful in building block for peptide modification [12], synthesis of pseudopeptides [13], and antimicrobial and antitumoral natural products [14]. Similarly, alkylated Meldrum's acid has been encountered in the synthesis of dehydro-ar-juvabione [15], indane subunit containing paraquinonic acid ethyl ester, and deliquinone natural products [16]. Thus, 2,2-dimethyl-1,3-dioxane-4,6-diones efficiently serves in the synthesis of versatile intermediates and for the synthesis of pharmacologically active molecules.

The recent literature survey reveals that nano-ZnO as heterogeneous catalyst has received considerable attention because of its ecofriendly nature and has been explored as a powerful catalyst for several organic transformations [17–22]. To the best of our knowledge, there is no report available in the literature regarding the reaction of 1*H*-indol-2,3-diones and activated methylene reagent (malononitrile/ethylcyanoacetate) with 2,2-dimethyl-1,3-dioxane-4,6-diones.

**Scheme 1:**
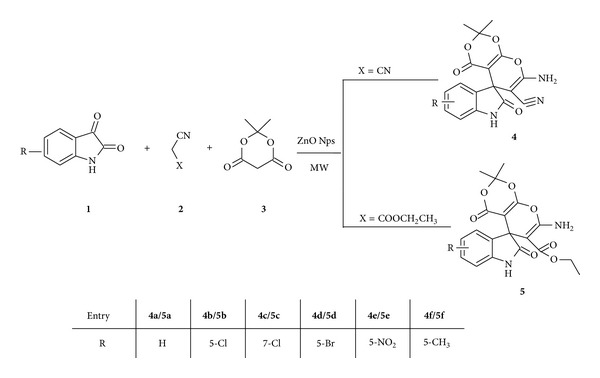


Hence, prompted by these observations and in continuation to our interest in organic synthesis by different methods with the use of nanocatalyst [23], we report an easy and rapid catalytic application of ZnO nanoparticles for one-pot synthesis of spiroindole derivatives incorporating pyranodioxine by the reaction of 1*H*-indole-2,3-dione, malononitrile/ethylcyanoacetate, and Meldrum's acid in absolute ethanol under microwave irradiation and conventional heating (Scheme 1). The overall process involves the Knoevenagel condensation of Meldrum's acid with 1*H*-indole-2,3-dione followed by “in situ” Michael addition of malononitrile/ethyl cyanoacetate in single operation to give spiro(indole-pyranodioxine) derivatives similar to the earlier reports [24, 25] of the formation of spiroindoles in the reaction of 3-carboethoxycyanomethylene-2*H*-indol-2-ones with cyclic ketones under classical conditions (Scheme 1). The process described here offers rapid facile one-pot synthesis of spiroindole derivatives using easily recyclable ZnO nanoparticles. This process is cost effective and hence ecofriendly as it is one-pot synthesis with easy workup and does not require harsh reagents. The process developed by us requires less quantity of catalyst (30 mg) for carrying out the catalytic reaction, thus decreasing the amount of effluent to considerable level.

## 2. Results and Discussion

The reaction of 1*H*-indole-2,3-dione **(1)**, malononitrile/ethylcyanoacetate **(2)**, and 2,2-dimethyl-1,3-dioxane-4,6-diones** (3)** was examined in the presence of catalytic amount (30 mg) of ZnO nanoparticle under microwave irradiation and conventional heating to give novel 7′-amino-2′2′-dimethyl-2,4′-dioxo-1,2-dihydrospiro[indoline-3,5′-pyrano[2,3-d][1′,3′]dioxine]-6′-carbonitrile/carboxyethylester **(4a–f)/(5a–f)** (Scheme 1) (Table 1).

To obtain the optimal conditions, the synthesis of **4a** and **5a** was used as a model reaction. A mixture of 1, 2, and 3 in the presence of ZnO nanoparticles (30 mg) was either refluxed for 10 hrs or irradiated inside microwave oven for 9 min resulting in the formation of **4a** in 62 or 87% yield, respectively (Table 2).

In order to confirm the effective involvement of ZnO nanoparticle during this transformation, a control experiment was conducted in the absence of ZnO nanoparticle for **4a**, the reaction did not proceed, and the substrate remained unchanged even after 35 minutes of microwave irradiation and 25 hrs of conventional heating (Table 2), while good results were obtained in the presence of ZnO nanoparticles. After some preliminary experiments, we found that a mixture of 1*H*-indole-2,3-dione, malononitrile, and 2,2-dimethyl-1,3-dioxane-4,6-diones in the presence of ZnO nanoparticle afforded products in 87% yield under microwave irradiation (Table 1).

Encouraged by these results, we have extended this reaction to variously substituted 1*H*-indole-2,3-diones under similar conditions to furnish the respective spiro(indole-pyranodioxine) derivatives in excellent yields (81–88%) using ZnO nanoparticle as a catalyst under microwave irradiation (Table 1).

Compounds were also synthesized under conventional heating using ZnO nanoparticle but yield of the product was found to be low (62–71%) as compared to that obtained under microwave irradiation. The synthesis of compound **4a** was carried out by refluxing for 10 hrs resulting in 62% yield, while under microwave irradiation, reaction took 9 min with 87% yield of the product. It showed that microwave irradiation was found to have a beneficial effect on the synthesis of spiro(indole-pyranodioxine) derivatives (Table 2).

On optimizing the amount of catalyst, we found that 30 mg of ZnO nanoparticles could effectively catalyze the reaction for the synthesis of desired product. With the inclusion of 10 mg and 20 mg, reaction took longer time. Using more than 30 mg has less effect on the yield and time of the reaction. Therefore, 30 mg of ZnO nanoparticles was sufficient to push the reaction forward, and further increasing of the amount of ZnO nanoparticles did not increase the yields (Table 3).

**Figure 1:**
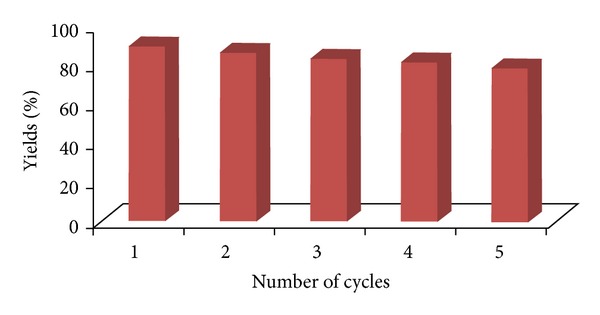
Recyclability of ZnO nanoparticles for the synthesis of 7′-amino-2′2′-dimethyl-2,4′dioxo-1,2-dihydrospiro[indoline-3,5′-pyrano[2,3-d][1′, 3′]dioxine]-6′-carbonitrile (**4a**).

Reusability is one of the most important properties of this catalyst. To study the recyclability of the catalyst, the ZnO nanoparticles were used for the same reaction repeatedly and the change in their catalytic activity was studied. The relation between the number of cycles of the reaction and the catalytic activity in terms of yield of product is presented in Figure 1. The catalyst recovered by filtration from the reaction mixture after dilution with ethyl acetate was reused as such for subsequent experiments under similar conditions. The catalyst retained optimum activity till five cycles after which drop in yield was observed (Figure 1).

A conceivable mechanism for the formation of the product would be as follows. The ZnO nanoparticle facilitate the Knoevenagel type coupling through Lewis acid sites (Zn^+2^) coordinated to the oxygen of carbonyl groups. On the other hand, ZnO nanoparticles can activate methylene compounds so that deprotonation of the C–H bond occurs in the presence of Lewis basic sites (O^−2^). As a result, the formation of spiroindole derivatives proceeds by activation of reactants through both Lewis acids and basic sites of ZnO nanoparticles.

## 3. Experimental

### 3.1. General

Reagents and solvents were obtained from commercial sources and used without further purification. Melting points were determined on a Toshniwal apparatus. The spectral analyses of synthesized compounds have been carried out at SAIF, Punjab University, Chandigarh. Purity of all compounds was checked by TLC using “G” coated glass plates and benzene : ethyl acetate (8 : 2) as eluent. IR spectra were recorded in KBr on a Perkin Elmer Infrared RXI FTIR spectrophotometer (Figure 3) and ^1^HNMR spectra were recorded on Bruker Avance II 400 NMR spectrometer using DMSO-d_6_ and CDCl_3_ as solvent and tetramethylsilane (TMS) as internal reference standard. The obtained products were identified from their spectral (^1^HNMR, ^13^C NMR, and IR) data. The microwave-assisted reactions were carried out in a Catalysts Systems Scientific Multimode MW oven attached with a magnetic stirrer and reflux condenser, operating at 700 W generating 2450 MHz frequency.

### 3.2. General Procedure for the Synthesis of ZnO Nanoparticle

Nanoparticles were synthesized by the literature method [26]. Zinc acetate dihydrate, sodium hydroxide, CTAB, and the other reagents used were all analytical grade (from Shanghai Chemical Corp.) without further purification and reactions were carried out in air. In a typical synthesis, zinc acetate dihydrate, CTAB, and sodium hydroxide were mixed (molar ratio 1 : 0.4 : 3) and ground together in an agate mortar for 50 min at room temperature (25°C). The reaction started readily during the mixing process, accompanied by the release of heat. The mixture was washed with distilled water in an ultrasonic bath. Finally, the product was dried in air at 60°C for 2 hrs. 

**Scheme 2:**
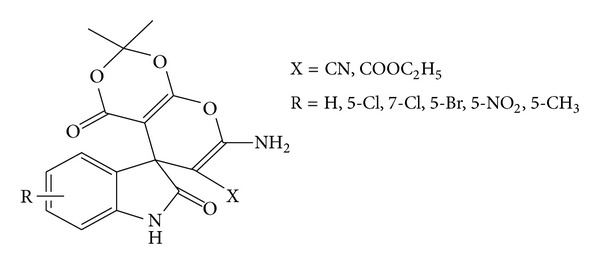


*Synthesis of 7′-Amino-2′2′-dimethyl-2, 4′dioxo-1,2-dihydrospiro[indoline-3,5′-pyrano[2,3-d]-[1′,3′]dioxine]-6′-carbonitrile/carboxyethylester (**4a**–**f**)/(**5a**–**f**) (see Scheme 2).* Compounds **4a–f** and **5a–f** were prepared by two different methods. 

*Method A: Microwave Irradiation Method*. An equimolar mixture of 1H-indole-2,3-dione **(1)** (1 mmole), malononitrile/ethylcynoacetate (1 mmole) **(2)**, and 2,2-dimethyl-1,3-dioxane-4,6-diones (1 mmole) **(3)** taken in absolute ethanol (15 mL) in presence of ZnO nanoparticle (30 mg) was charged into a glass microwave vessel and refluxed inside a microwave oven at 420 watts for 9-10 min. Progress of the reaction was monitored by TLC. After completion of reaction, the reaction mixture was cooled to room temperature and solidified within an hour. The resulting solidified mixture was diluted with ethyl acetate (5 mL) and the catalyst was separated. The filtrate was evaporated on rota-evaporator to give a solid, which was dried and recrystallized from ethyl acetate. 

*Method B: Conventional Heating Method*. An equimolar mixture of 1H-indole-2,3-dione **(1)** (1 mmole), malononitrile/ethylcynoacetate **(2)** (1 mmole), and 2,2-dimethyl-1,3-dioxane-4,6-diones** (3)** (1 mmole) taken in absolute ethanol (15 mL) in presence of ZnO nanoparticle (30 mg) was refluxed for 10-11 hrs Progress of the reaction was monitored by TLC. After completion of reaction, the reaction mixture was cooled to room temperature and solidified within an hour. The resulting solidified mixture was diluted with ethyl acetate (5 mL) and the catalyst was separated. The filtrate was evaporated on rota-evaporator to give a solid, which was dried and recrystallized from ethyl acetate.

All the synthesized compounds were identified by their melting point, IR, ^1^HNMR, ^13^CNMR, and mass spectral studies.

The spectroscopic characterization data of (**4a–f/5a–f**) are given below. 

***4a**  7′-Amino-2′2′-dimethyl-2,4′-dioxo-1,2-dihydrospiro[indoline-3,5′-pyrano[2,3-d][1′,3′]dioxine]-6′-carbonitrile.* IR (cm^−1^): 3400 (NH_2_), 3265 (NH), 3059 (aromatic C–H str), 2984 (aliphatic C–H str), 2204 (CN), 1727 (NH–C=O), 1620 (O=C–O), 1188.46 (C–O–C); ^1^HNMR (*δ* ppm): 10.93 (s, 1H, NH indole), 7.89 (s, 2H, NH_2_), 6.88–8.22 (m, 4H, Ar–H), 1.48 (s, 3H, CH_3_), 1.90 (s, 3H, CH_3_); ^13^CNMR (*δ* ppm): 166.4 (C=O), 159.4 (C–NH_2_), 124.0–141.6 (aromatic carbons), 117.1 (C*≡*N), 110.1 (O–C–O), 46.6 (Spiro carbon), 26.3 (CH_3_), 24.1 (CH_3_); Anal. Calcd. for C_17_H_13_N_3_O_5_: C, 60.1, H, 3.8, N, 12.3. Found: C, 60.01, H, 3.85, N, 12.37; MS: [M]^+^ at *m*/*z* 339. 

***4b**  7′-Amino-2′2′-dimethyl-2,4′-dioxo-5-chloro-1,2-dihydrospiro[indoline-3,5′-pyrano[2,3-d][1′,3′]dioxine]-6′-carbonitrile.* IR (cm^−1^): 3410 (NH_2_), 3260 (NH), 3053 (aromatic C–H str), 2980 (aliphatic C–H str), 2202 (CN), 1720 (NH–C=O), 1622 (O=C–O), 1180 (C–O–C); ^1^HNMR (*δ* ppm): 10.84 (s, 1H, NH indole), 7.78 (s, 2H, NH_2_), 6.82–8.20 (m, 3H, Ar–H), 1.42 (s, 3H, CH_3_), 1.94 (s, 3H, CH_3_); ^13^CNMR (*δ* ppm): 166.4 (C=O), 159.4 (C–NH_2_), 124.0–141.6 (aromatic carbons), 117.1 (C*≡*N), 110.1 (O–C–O), 46.6 (Spiro carbon), 26.3 (CH_3_), 24.1 (CH_3_); Anal. Calcd. for C_17_H_12_ClN_3_O_5_: C, 54.63, H, 3.24, N, 11.24. Found: C, 54.80, H, 3.26, N, 11.26; MS: [M]^+^ at *m/z* 373.74. 

***4c**  7′-Amino-2′2′-dimethyl-2,4′-dioxo-7-chloro-1,2-dihydrospiro[indoline-3,5′-pyrano[2,3-d][1′,3′]dioxine]-6′-carbonitrile.* IR (cm^−1^): 3402 (NH_2_), 3256 (NH), 3039 (aromatic C–H str), 2977 (aliphatic C–H str), 2210 (CN), 1732 (NH–C=O), 1610 (O=C–O), 1175 (C–O–C); ^1^HNMR (*δ* ppm): 10.78 (s, 1H, NH indole), 7.76 (s, 2H, NH_2_), 6.84–8.26 (m, 3H, Ar–H), 1.46 (s, 3H, CH_3_), 1.92 (s, 3H, CH_3_); ^13^CNMR (*δ* ppm): 166.4 (C=O), 159.4 (C–NH_2_), 124.0–141.6 (aromatic carbons), 117.1 (C*≡*N), 110.1 (O–C–O), 46.6 (Spiro carbon), 26.3 (CH_3_), 24.1 (CH_3_); Anal. Calcd. for C_17_H_12_ClN_3_O_5_: C, 54.63, H, 3.24, N, 11.24. Found: C, 54.79, H, 3.27, N, 11.26; MS: [M]^+^ at *m/z* 373.74. 

***4d**  7′-Amino-2′2′-dimethyl-2,4′-dioxo-5-bromo-1,2-dihydrospiro[indoline-3,5′-pyrano[2,3-d][1′,3′]dioxine]-6′-carbonitrile.* IR (cm^−1^): 3400 (NH_2_), 3280 (NH), 3050 (aromatic C–H str), 2974 (aliphatic C–H str), 2200 (CN), 1722 (NH–C=O), 1618 (O=C–O), 1180 (C–O–C); ^1^HNMR (*δ* ppm): 10.74 (s, 1H, NH indole), 7.72 (s, 2H, NH_2_), 6.87–8.26 (m, 3H, Ar–H), 1.40 (s, 3H, CH_3_), 1.98 (s, 3H, CH_3_); ^13^CNMR (*δ* ppm): 166.4 (C=O), 159.4 (C–NH_2_), 124.0–141.6 (aromatic carbons), 117.1 (C*≡*N), 110.1 (O–C–O), 46.6 (Spiro carbon), 26.3 (CH_3_), 24.1 (CH_3_); Anal. Calcd. for C_17_H_12_BrN_3_O_5_: C, 48.82, H, 2.89, N, 10.05. Found: C, 48.65, H, 2.91, N, 10.07; MS: [M]^+^ at *m/z* 418. 

***4e**  7′-Amino-2′2′-dimethyl-2,4′-dioxo-5-nitro-1,2-dihydrospiro[indoline-3,5′-pyrano[2,3-d][1′,3′]dioxine]-6′-carbonitrile.* IR (cm^−1^): 3400 (NH_2_), 3265 (NH), 3059 (aromatic C–H str), 2984 (aliphatic C–H str), 2204 (CN), 1727 (NH–C=O), 1620 (O=C–O), 1188.46 (C–O–C); ^1^HNMR (*δ* ppm): 10.78 (s, 1H, NH indole), 7.71 (s, 2H, NH_2_), 6.83–8.28 (m, 3H, Ar–H), 1.48 (s, 3H, CH_3_), 1.90 (s, 3H, CH_3_); ^13^CNMR (*δ* ppm): 166.4 (C=O), 159.4 (C–NH_2_), 124.0–141.6 (aromatic carbons), 117.1 (C*≡*N), 110.1 (O–C–O), 46.6 (Spiro carbon), 26.3 (CH_3_), 24.1 (CH_3_); Anal. Calcd. for C_17_H_12_N_4_O_7_: C, 53.13, H, 3.15, N, 14.58. Found: C, 53.30, H, 3.17, N, 14.59; MS: [M]^+^ at *m/z* 384. 

***4f**  7′-Amino-2′2′-dimethyl-2,4′-dioxo-5-methyl-1,2-dihydrospiro[indoline-3,5′-pyrano[2,3-d][1′,3′]dioxine]-6′-carbonitrile.* IR (cm^−1^): 3405 (NH_2_), 3261 (NH), 3048 (aromatic C–H str), 2981 (aliphatic C–H str), 2206 (CN), 1724 (NH–C=O), 1618 (O=C–O), 1178 (C–O–C); ^1^HNMR (*δ* ppm): 10.81 (s, 1H, NH indole), 7.75 (s, 2H, NH_2_), 6.81–8.20 (m, 3H, Ar–H), 2.35 (s, 3H, CH_3_) 1.40 (s, 3H, CH_3_), 1.98 (s, 3H, CH_3_); ^13^CNMR (*δ* ppm): 166.4 (C=O), 159.4 (C–NH_2_), 124.0–141.6 (aromatic carbons), 117.1 (C*≡*N), 110.1 (O–C–O), 46.6 (Spiro carbon), 26.3 (CH_3_), 24.1 (CH_3_), 25.1 (CH_3_); Anal. Calcd. for C_18_H_15_N_3_O_5_: C, 61.19, H, 4.28, N, 11.89. Found: C, 61.40, H, 4.30, N, 11.87; MS: [M]^+^ at *m/z* 353. 

***5a**  7′-Amino-2′2′-dimethyl-2,4′-dioxo-1,2-dihydrospiro[indoline-3,5′-pyrano[2,3-d][1′,3′]dioxine]-6′-carboxyethylester.* IR (cm^−1^): 3402 (NH_2_), 3298 (NH), 3059 (aromatic C–H str), 2984 (aliphatic C–H str), 1727 (NH–C=O), 1620 (O=C–O), 1184 (C–O–C); ^1^HNMR (*δ*) 10.97 (s, 1H, NH indole), 8.15 (s, 2H, NH_2_), 6.92–7.46 (m, 4H, Ar–H), 4.44 (q, 2H, CH_2_), 1.35 (t, 3H, CH_3_), 1.18 (s, 3H, CH_3_), 1.14 (s, 3H, CH_3_); ^13^CNMR (*δ*) 167.7 (O–C–O), 166.4 (NH–C=O), 166.1 (C=O), 159.2 (C–NH_2_), 124.3–142.0 (aromatic carbons), 61.2 (OCH_2_), 46.6 (spiro carbon), 26.3 (CH_3_), 24.1 (CH_3_), 14.1 (CH_3_ ester); Anal. Calcd. for C_19_H_18_N_2_O_7_: C, 59.07, H, 4.70, N, 7.25. Found: C, 59.25, H, 4.68, N, 7.26; MS: [M]^+^ at *m/z* 386. 

***5b**  7′-Amino-2′2′-dimethyl-2,4′-dioxo-5-chloro-1,2-dihydrospiro[indoline-3,5′-pyrano[2,3-d][1′,3′]dioxine]-6′-carboxyethylester.* IR (cm^−1^): 3400 (NH_2_), 3292 (NH), 3050 (aromatic C–H str), 2980 (aliphatic C–H str), 1722 (NH–C=O), 1622 (O=C–O), 1182 (C–O–C); ^1^HNMR (*δ*): 10.90 (s, 1H, NH indole), 8.12 (s, 2H, NH_2_), 6.92–7.26 (m, 3H, Ar–H), 4.40 (q, 2H, CH_2_), 1.32 (t, 3H, CH_3_), 1.12 (s, 3H, CH_3_), 1.10 (s, 3H, CH_3_); ^13^CNMR (*δ*): 167.7 (O–C–O), 166.4 (NH–C=O), 166.1 (C=O), 159.2 (C–NH_2_), 124.3–142.0 (aromatic carbons), 61.2 (OCH_2_), 46.6 (spiro carbon), 26.3 (CH_3_), 24.1 (CH_3_), 14.1 (CH_3_ ester); Anal. Calcd. for C_19_H_17_ ClN_2_O_7_: C, 54.23, H, 4.07, N, 6.66. Found: C, 54.40, H, 4.05, N, 6.65; MS: [M]^+^ at *m/z* 420.80. 

***5c**  7′-Amino-2′2′-dimethyl-2,4′-dioxo-7-chloro-1,2-dihydrospiro[indoline-3,5′-pyrano[2,3-d][1′,3′]dioxine]-6′-carboxyethylester.* IR (cm^−1^): 3408 (NH_2_), 3290 (NH), 3058 (aromatic C–H str), 2984 (aliphatic C–H str), 1728 (NH–C=O), 1624 (O=C–O), 1188 (C–O–C); ^1^HNMR (*δ*): 10.92 (s, 1H, NH indole), 8.10 (s, 2H, NH_2_), 6.90–7.20 (m, 3H, Ar–H), 4.42 (q, 2H, CH_2_), 1.30 (t, 3H, CH_3_), 1.16 (s, 3H, CH_3_), 1.10 (s, 3H, CH_3_); ^13^CNMR (*δ*): 167.7 (O–C–O), 166.4 (NH–C=O), 166.1 (C=O), 159.2 (C–NH_2_), 124.3–140.0 (aromatic carbons), 61.2 (OCH_2_), 46.6 (Spiro carbon), 26.3 (CH_3_), 24.1 (CH_3_), 14.1 (CH_3_ ester); Anal. Calcd. for C_19_H_17_ ClN_2_O_7_: C, 54.23, H, 4.07, N, 6.66. Found: C, 54.42, H, 4.06, N, 6.64; MS: [M]^+^ at *m/z* 420.80. 

***5d**  7′-Amino-2′2′-dimethyl-2,4′-dioxo-5-bromo-1,2-dihydrospiro[indoline-3,5′-pyrano[2,3-d][1′,3′]dioxine]-6′-carboxyethylester.* IR (cm^−1^): 3400 (NH_2_), 3260 (NH), 3050 (aromatic C–H str), 2982 (aliphatic C–H str), 1790 (C=O), 1722 (NH–C=O), 1618 (O=C–O), 1130 (C–O–C); ^1^HNMR (*δ*): 10.74 (s, 1H, N indole), 6.90 (s, 2H, NH_2_), 6.87–8.26 (m, 3H, Ar–H), 4.19 (q, 2H, CH_2_), 1.98 (s, 3H, CH_3_), 1.40 (s, 3H, CH_3_), 1.34 (t, 3H, CH_3_); ^13^CNMR (*δ*): 167.7 (O–C–O), 166.4 (NH–C=O), 166.1 (C=O), 159.2 (C–NH_2_), 124.3–142.0 (aromatic carbons), 61.2 (OCH_2_), 46.6 (Spiro carbon), 26.3 (CH_3_), 24.1 (CH_3_), 14.1 (CH_3_ ester); Anal. Calcd. for C_19_H_17_ BrN_2_O_7_: C, 49.05, H, 3.68, N, 6.02. Found: C, 49.24, H, 3.70, N, 6.04; MS: [M]^+^ at *m/z* 465. 

***5e**  7′-Amino-2′2′-dimethyl-2,4′-dioxo-5-nitro-1,2-dihydrospiro[indoline-3,5′-pyrano[2,3-d][1′,3′]dioxine]-6′-carboxyethylester.* IR (cm^−1^): 3420 (NH_2_), 3290 (NH), 3069 (aromatic C–H str), 1732 (NH–C=O), 1620 (O=C–O), 1180 (C–O–C); ^1^HNMR (*δ*): 10.92 (s, 1H, NH indole), 8.14 (s, 2H, NH_2_), 7.2–8.08 (m, 3H, Ar–H), 4.40 (q, 2H, CH_2_), 2.78 (s, 1H, NH), 1.38 (t, 3H, CH_3_), 1.16 (s, 3H, CH_3_), 1.10 (s, 3H, CH_3_); ^13^CNMR (*δ*): 167.7 (O–C–O), 166.4 (NH–C=O), 166.1 (C=O), 159.2 (C–NH_2_), 124.3–142.0 (aromatic carbons), 61.2 (OCH_2_), 46.6 (Spiro carbon), 26.3 (CH_3_), 24.1 (CH_3_), 14.1 (CH_3_ ester); Anal. Calcd. for C_19_H_17_ N_3_O_9_: C, 52.90, H, 3.97, N, 9.74. Found: C, 52.71, H, 3.95, N, 9.75; MS: [M]^+^ at *m/z* 431. 

***5f**  7′-Amino-2′2′-dimethyl-2,4′-dioxo-5-methyl-1,2-dihydrospiro[indoline-3,5′-pyrano[2,3-d][1′,3′]dioxine]-6′-carboxyethylester.* IR (cm^−1^): 3434 (NH_2_), 3298 (NH), 3060 (aromatic C–H str), 1730 (NH–C=O), 1628 (O=C–O), 1188 (C–O–C); ^1^HNMR (*δ*): 10.90 (s, 1H, NH indole), 8.12 (s, 2H, NH_2_), 6.92–7.26 (m, 3H, Ar–H), 4.40 (q, 2H, CH_2_), 2.32 (s, 3H, CH_3_), 1.32 (t, 3H, CH_3_), 1.12 (s, 3H, CH_3_), 1.10 (s, 3H, CH_3_); ^13^CNMR (*δ*): 167.7 (O–C–O), 166.4 (NH–C=O), 166.1 (C=O), 159.2 (C–NH_2_), 124.3–142.0 (aromatic carbons), 61.2 (OCH_2_), 46.6 (spiro carbon), 26.3 (CH_3_), 24.1 (CH_3_), 14.1 (CH_3_ ester); Anal. Calcd. for C_20_H_20_ N_2_O_7_: C, 60.00, H, 5.03, N, 7.00. Found: C, 60.19, H, 5.05, N, 6.99; MS: [M]^+^ at *m/z* 400.

### 3.3. Characterization of the Synthesized ZnO Nanoparticles

The synthesized ZnO nanoparticles were characterized by using X-ray diffraction (XRD), FTIR, UV-VIS spectra, and fluorescence spectroscopy.

#### 3.3.1. XRD Pattern of ZnO Nanoparticles

**Figure 2:**
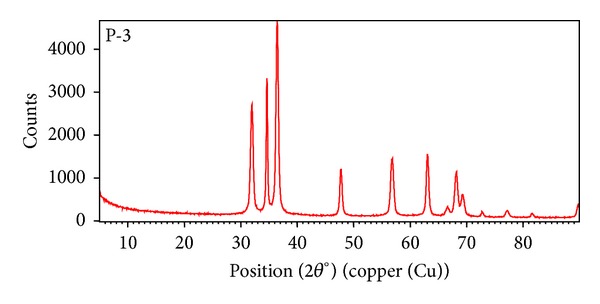
XRD pattern of ZnO nanoparticles.

The nanostructure of ZnO nanoparticle has been studied at room temperature by using X-ray diffraction pattern (Figure 2). The particle size was calculated from X-ray diffraction images of ZnO powders using the Scherrer formula:

(1) $$\begin{array}{c} D=\frac{K\lambda }{\beta \cos\theta }, \end{array}$$

where *D* is the average particle size perpendicular to the reflecting planes, *λ* is the X-ray wavelength, *β* is the full width at half maximum (FWHM), and *θ* is the diffraction angle. The average size of ZnO nanoparticles obtained from the XRD is about 5.1 nm, using the Scherrer formula.

#### 3.3.2. Fourier Transforms Infrared Spectroscopy (FTIR)

**Figure 3:**
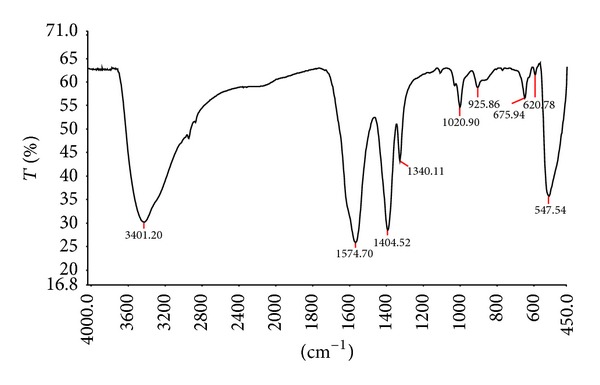
FTIR of ZnO nanoparticles.

The FTIR was acquired in the range of 400–4000 cm^−1^ (Figure 3). The band between 450–550 cm^−1^ correlated to metal oxide bond (ZnO). The peaks in the range 1400–1500 cm^−1^ correspond to CO bonds. The peaks at 1340 cm^−1^ and 1574 cm^−1^ correspond to CO and OH bending vibrations, respectively. IR spectra were recorded in KBr on a Perkin Elmer Infrared RXI FTIR spectrophotometer.

#### 3.3.3. U-V Spectroscopy

**Figure 4:**
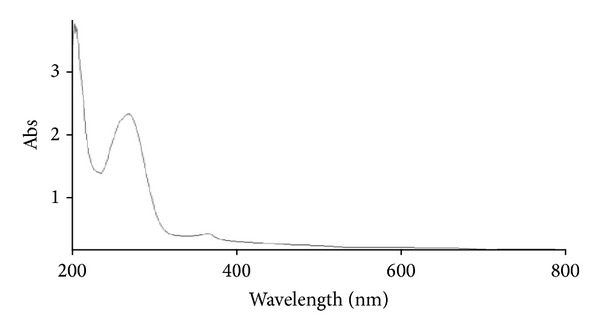
U-V spectrum of ZnO nanoparticles.

The U-V spectrum was taken by using Cary 60 UV-VIS, Agilent Technologies. The sample was vigorously mixed through vortex for 10 min. The U-V absorption spectrum of ZnO nanoparticle in methanol gave absorption peak at 275 nm (Figure 4).

#### 3.3.4. Fluorescence Spectroscopy

**Figure 5:**
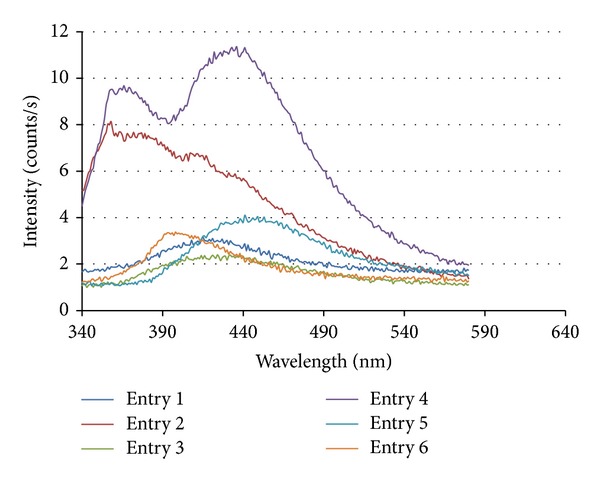
Fluorescence spectrum of ZnO nanoparticles.

The fluorescence spectrum of nano-ZnO at different molar concentrations in methanol was taken at different excitation wavelength 300–600 nm (Figure 5) (Table 4). All the samples were vigorously mixed through vortex for 10 min. Fluorescence spectrum was recorded by using spectrofluorophotometer model number 5301PC, Shimadzu Cooperation, Kyoto, Japan.

## 4. Conclusion

We have demonstrated an environ-economic and simple protocol for the synthesis of novel spiroindole derivatives by the one-pot three-component reaction of isatin, malononitrile/ethylcyanoacetate, and Meldrum's acid with ZnO nanoparticles as a green, effective, and recoverable catalyst. The catalyst can be recycled and reused without apparent loss of activity.

## Figures and Tables

**Table 1:** Experimental data of  7′-amino-2′2′-dimethyl-2,4′dioxo-1,2-dihydrospiro[indoline-3,5′-pyrano[2,3-d]-1′,3′dioxine]-6′-carbonitrile (**4a**–**f**)/carboxyethylester (**5a**–**f**) under microwave irradiation (Method A) and conventional heating (Method B).

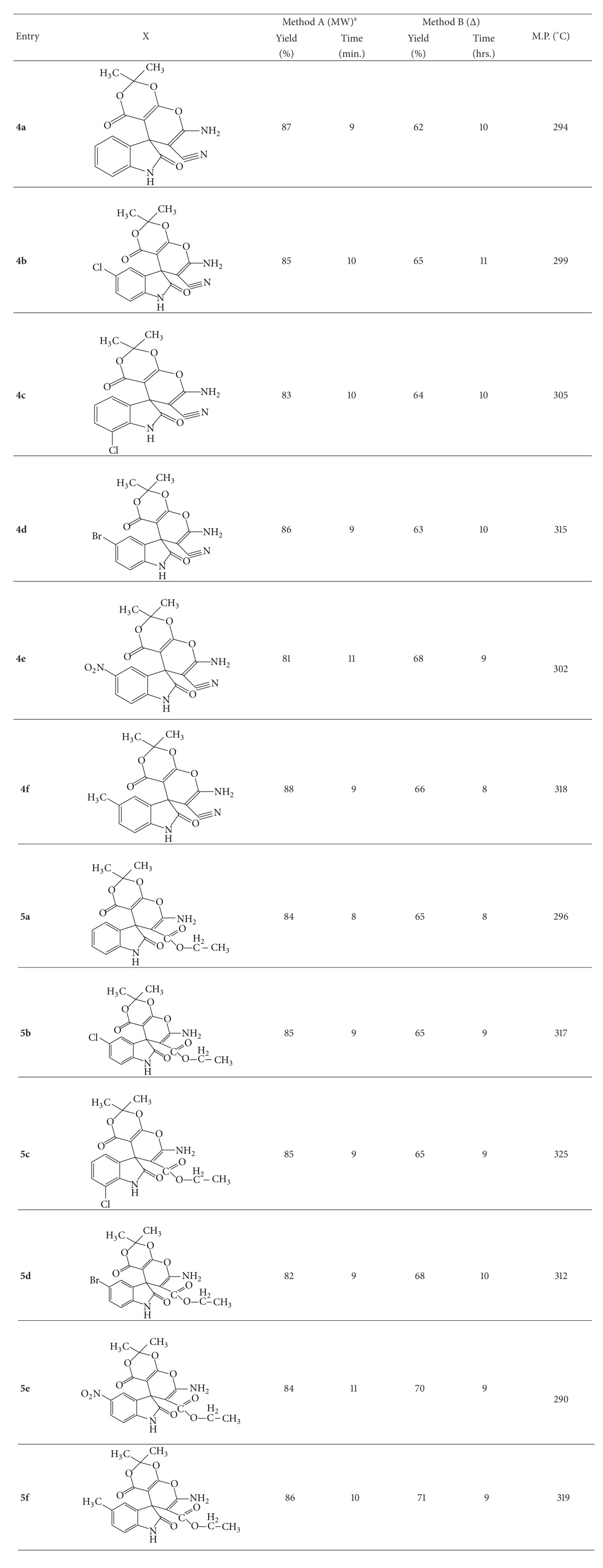

Reaction conditions: Meldrum's acid (1.0 mmol), isatin (1.0 mmol), malononitrile/ethylcyanoacetate (1.0 mmol), absolute ethanol (15 mL), and catalyst (30 mg). ^a^Reaction under microwave irradiation was carried out at 420 watts.

**Table 2:** Comparison of catalytic activity of ZnO nanoparticles in the synthesis of compounds **4a** and **5a** by conventional heating (Δ) and microwave irradiation method (MW).

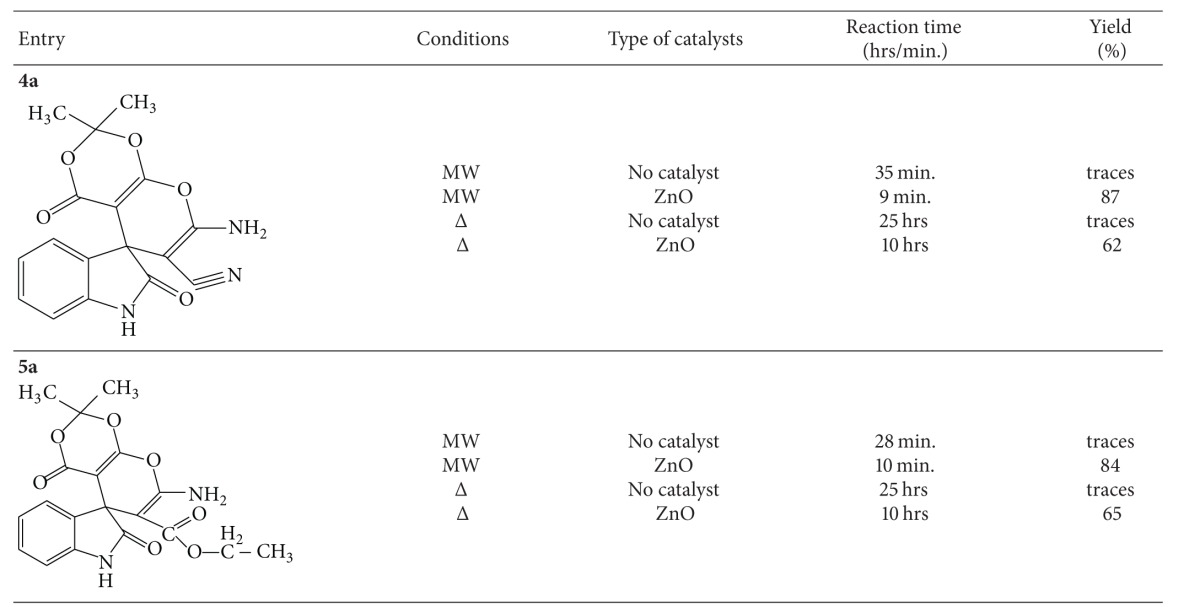

Amount of Reactants: Meldrum acid (1.0 mmol), isatin (1.0 mmol), and malononitrile/ethylcyanoacetate

(1.0 mmol); reaction under microwave irradiation was carried out at 420 watts.

**Table 3:** Optimization of the ZnO nanoparticle catalyzed model reaction for synthesis of 7′-amino-2′2′-dimethyl-2,4′-dioxo-1,2-dihydrospiro[indoline-3,5′-pyrano[2,3-d][1′,3′]dioxine]-6′-carbonitrile (**4a**).

Entry	Catalyst (mg)	Yield (%)
1	No catalyst	—
2	10	80
3	15	81
4	20	83
5	25	86
6	30	89

Amount of Reactants: Meldrum acid (1.0 mmol), isatin (1.0 mmol), malononitrile (1.0 mmol), and absolute ethanol (15 mL).

**Table 4:** Fluorescence data ZnO in methanol at different molar concentrations.

Entry	Molar concentration	ZnO nanoparticle
		*λ*em (nm)
1	1 × 10^−5^	420
2	2 × 10^−5^	280
3	3 × 10^−5^	395
4	4 × 10^−5^	445, 365
5	5 × 10^−5^	455
6	6 × 10^−5^	400
